# Income Support as a Health Intervention

**DOI:** 10.1001/jamanetworkopen.2021.43363

**Published:** 2022-01-04

**Authors:** Heather D. Hill, Ali Rowhani-Rahbar

**Affiliations:** Daniel J. Evans School of Public Policy & Governance, University of Washington, Seattle; Department of Epidemiology, School of Public Health, University of Washington, Seattle

The increasing recognition of social and economic factors as fundamental to health and health disparities has dramatically broadened the topics, levels of analysis, and interventions considered relevant to public health and medicine. The article by Shafer and colleagues^1^ is an excellent example of this broader orientation. The authors examined the short-term associations of giving most US families with children an advanced monthly payment on a substantial tax credit with food insufficiency. Household food insufficiency, defined as not having enough to eat, is a material hardship associated with many adverse health outcomes. The policy they studied, the Child Tax Credit (CTC), was not designed to address food insufficiency or health per se but to offset the substantial costs of providing children with high-quality housing, nutrition, education, and more. The authors found that the first CTC payment—ranging from $250 to many times that, depending on the number and ages of children—was associated with a 26% reduction in food insufficiency nationally among households with children.^1^

We are living through a wave of experimentation with new forms of income support, ie, programs that provide income directly to individuals and families. Active innovation in this policy area—at local, state, and federal levels—grew from the convergence of scientific evidence on child poverty,^2^ renewed attention to structural causes of poverty, and the economic crisis associated with the COVID-19 pandemic. The federal CTC expansion studied by Shafer et al^1^ is the highest profile of these innovations. Originally a program designed to reduce middle-income families’ tax liability, the CTC did not benefit low-income families with little or no tax liability. The American Rescue Plan Act of 2021 expanded the program temporarily by making it refundable (ie, payable in cash, not just as a reduction of tax liability) and increasing its value. In doing so, Congress created the closest thing that the United States has ever had to a child allowance, a universal public income guarantee to offset the costs of raising children.

There are many examples of smaller, more tailored income support programs at local and state levels, spurring randomized controlled trials (RCTs) and natural experiments examining health outcomes. For example, Baby’s First Years is an RCT testing the effect of unconditional income support in 4 sites around the country. Mothers were recruited at the time of a birth and randomized to a sizable ($333) or small ($20) monthly income supplement. The interdisciplinary research team will collect data on a range of outcomes, including maternal and child health and brain development, every 12 months until the children are aged 4 years.^3^

Many cities are also partnering with philanthropy to pilot basic income programs, in which low-income families receive a monthly stipend with no requirements for specific behaviors, such as working. The first such program, the Stockton Economic Empowerment Demonstration (SEED), provided $500 per month for 24 months to 125 residents of Stockton, California, living in low-income neighborhoods. Positive results from an RCT evaluation of SEED—including reductions in maternal depression and anxiety—quickly inspired more than 2 dozen pilot programs in cities as diverse as Denver, Colorado; Columbia, South Carolina; and Tacoma, Washington.^4^

Increased recognition of the harm done by racist policies of enslavement, erasure, and exclusion adds to our understanding of how economic and racial and ethnic inequities interact to influence health and how targeted reparations might tackle multiple causes of health disparities. Eleven US mayors have committed to piloting reparations programs for African Americans, which could eventually provide cash payments, community investments, or social programs.^5^ Once implemented, these programs will offer insight into the ability to address the historical roots of health disparities through modern-day income support and investments. As Shafer et al ^1^ note, programs like the CTC, which are built on structurally unequal systems (eg, the labor market and the tax system), run the risk of reproducing rather than redressing racial and ethnic health disparities if their design and implementation are not intentional.

The study by Shafer et al^1^ adds evidence to current policy discussions about maintaining the expansion of the CTC. More broadly, the study suggests that the production and promotion of health may be best achieved not by exclusively investing in programs and practices designed to address disease per se but also in broader economic and social policies that target its fundamental causes. Generating evidence on the health-related outcomes of a range of such programs and policies is critically needed. For instance, in the past 3 years, we have been part of a team studying the Earned Income Tax Credit (EITC), a tax credit for workers with low earnings and children in the home, and a range of health and violence outcomes. Our research used 12 data sources and, like Shafer et al,^1^ statistical techniques that harness the natural experiment of policy changes. We found that more generous state EITCs were associated with reduced rates of child maltreatment and adult mental distress, particularly among those with lower educational attainment, who are more likely to receive the EITC.^6,7^

There is still much to learn about how best to use income support as a health intervention. We hope current and future researchers will be inspired by Shafer et al^1^ to study the CTC and other innovations in income support over time and in relation to multiple domains of child and adult health. The current wave of new and expanded income support programs offers an enormous opportunity to health researchers to better understand how economic hardship has both short-term and lifelong effects on our physiology and psychology. In return, the scientific evidence offers advocates, policy makers, and program administrators insights into designing income support to maximize its health-promoting effects.
